# Non-operative management of appendiceal abscess in children: a systematic review

**DOI:** 10.1080/07853890.2025.2566875

**Published:** 2025-09-30

**Authors:** Song Zhang, Hai Lan Zhang, Yu Zuo Bai, Xiao Bing Tang

**Affiliations:** Department of Pediatric Surgery, Shengjing Hospital of China Medical University, Shenyang, China

**Keywords:** Appendiceal abscess, non-operative treatment, active observation, Interval appendectomy, children

## Abstract

**Background:**

Non-operative treatment (NOT) has gained widespread acceptance as the initial therapy for pediatric patients with appendiceal abscess. The subsequent management methods encompass interval appendectomy (IA) and active observation (AO). The aim of the study is to compare the IA and AO alternatives after successful NOT of pediatric appendiceal abscess.

**Methods:**

Systematic literature research of PubMed was performed for relevant studies published. Variables as length of hospital stay (LOS), recurrence rate, complication rate, cost, operative time were analyzed.

**Results:**

Six articles were selected, including 396 patients with successful NOT of appendiceal abscess. 145 patients were in IA group while 251 patients were in AO group. There was no statistically significant difference in complication rate and cost was observed between IA and AO with recurrence group, while IA resulted in a shorter LOS and operative time. Moreover, the incidence rate of recurrence in AO was reported as 2.4%∼34.2%. The pooled estimate of the risk of recurrence was 9.9% (CI:4.8–20.6).

**Conclusion:**

Following successful NOT of pediatric appendiceal abscess, the choice of management options is still controversial. More well-designed randomized controlled trials are needed to discover optimal treatment strategy.

## Introduction

In the pediatric population, acute appendicitis is the most common gastrointestinal surgical disease with a cumulative lifetime risk of 7%−8% [1,2]. An appendiceal abscess is the localized collection of pus secondary to perforation of an acutely inflamed appendix, confined by inflammatory adhesions from adjacent abdominal structures [3–5]. Based on the accumulating evidences, the application of initial non-operative treatment (NOT) has been regarded as the primary approach and achieved effective therapeutic outcomes in cases of appendiceal abscess [6–9].

The subsequent management methods after the success of NOT include interval appendectomy (IA) and active observation (AO). IA is usually performed after 8 to 12 weeks from symptom resolution [3]. Besides, AO is scheduled regular review during the follow-up period [10].

Nowadays, the choice of IA or AO after NOT is still a controversial issue. Nonetheless, there were hardly any systematic reviews about the comparison with the management after successful NOT. Therefore, the aim of this paper is to systematically review the available literature and compare the outcomes of interventions utilizing IA and AO.

## Method

To conduct this systematic review, the Preferred Reporting Items for Systematic Reviews and Meta-analyses (PRISMA) standard version 2020 were followed[11]. This review was registered on PROSPERO (CRD420251112940).

## Search strategy

**Figure 1.**
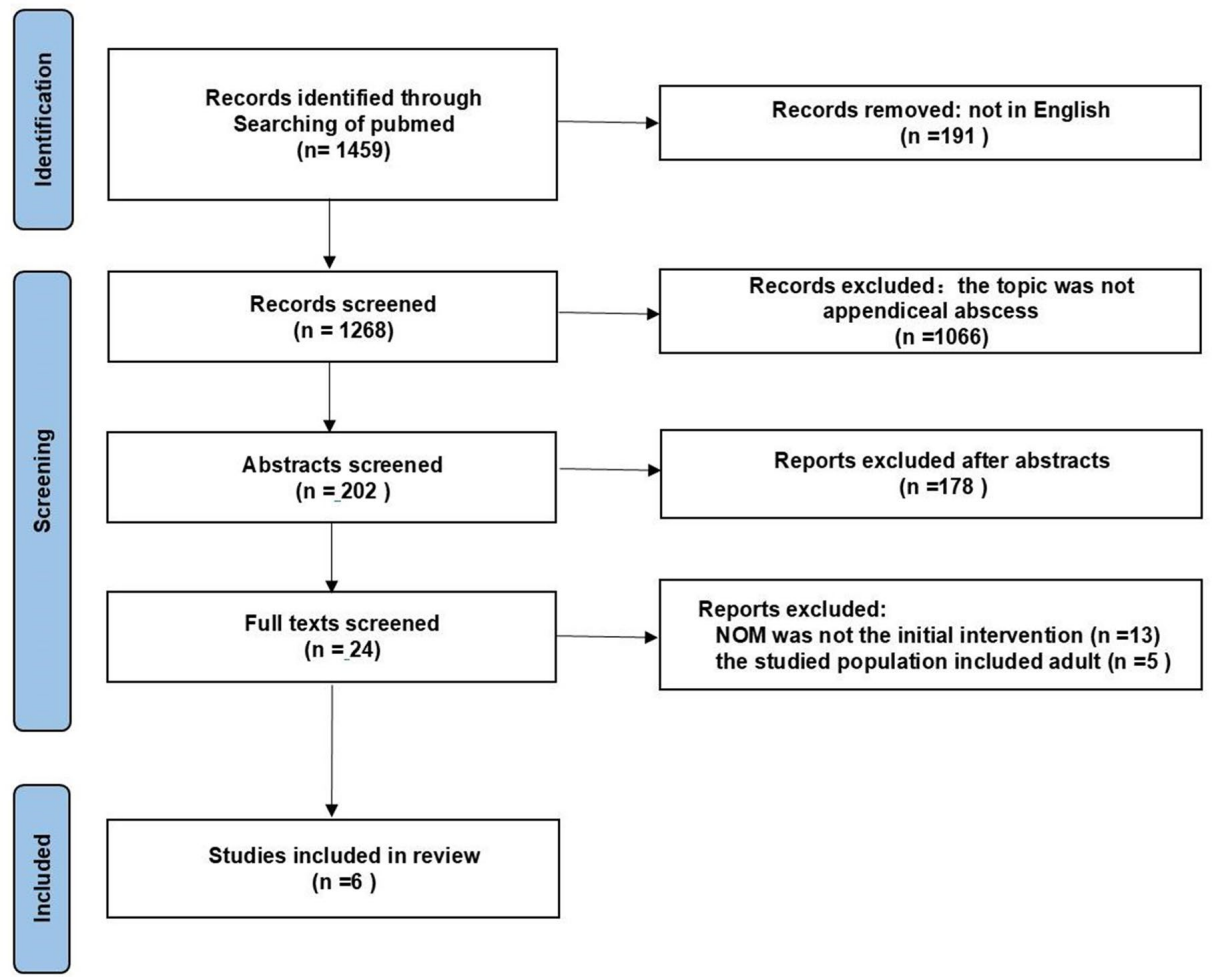
PRISMA flow diagram of study selection.

A systematic search was performed on PubMed with the profiles: ‘appendiceal abscess OR appendiceal mass’. These sources were searched up to December 31, 2023 and 1459 references were identified. We included 1268 records of different study designs with prospective, randomized, nonrandomized and observational, prospective and retrospective studies that reported in English. According to title and abstract, the retrieved articles were preliminary screened and the articles whose topic was not appendiceal abscess were excluded. Subsequently, the full text was analyzed to identify original studies is associated with non-operative management of appendiceal abscess in children or not (Figure 1).

## Inclusion and exclusion criteria

Studies were included in this study that focused on comparing children with appendiceal abscess treated IA and AO after initial NOT. Studies were excluded if they were non-primary research, or if NOT was not the initial intervention, appendectomy for recurrent appendicitis was not separated from IA.

## Data extraction

The data were extracted independently by SZ and HLZ by using a standardized extraction form designed for this systematic review. The following information was extracted from individual articles: author’s name, publication year, publication country, type of study design, respondent’s information, sample size, statistical analysis used and outcomes measures by the studies. Outcome Measures as length of hospital stay (LOS), recurrence rate, complication rate, cost, and operative time were reviewed.

## Data analysis

A meta-analysis was performed for the recurrence rate using RevMan 5.4 software (Cochrane Collaboration, Oxford, UK). The recurrence rates from the individual studies were pooled by using a random-effect meta-analysis. The *P*-value for statistical significance was set at 0.05.

## Results

Figure 1 summarizes the results of the search and selection of articles. Based on the criteria, 6 articles were incorporated in the review, including 1 prospective randomized control trial (RCT) [10], 3 prospective cohort studies [12–14]. 1 retrospective cohort studies [15], and 1 case control study [16]. The general characteristics of the included studies were shown in Table 1. 396 patients with appendiceal abscess who underwent successful NOT were enrolled in these studies. Among the included patients, 145 (36.6%) underwent IA after initial NOT, while 251 (63.4%) chose AO with regular follow-up (Table 1).

**Table 1.** Characteristics of 6 articles included in this review.

Study	Year	Country	Total	Age (year)	IA	Mean of Interval time (day)	AO	Recurrence	Follow-up time	Average time of recurrence	Surgery for recurrence
Hall et al. [10]	2017	UK	102	3–15	50	66	52	6 (12%)	1 year	within 1 year	6 (100%)
Tanaka et al. [12]	2016	Japan	54	4.1–15.8	16	90	38	13 (34.2%)	3.4 years	12.6 ± 22.3 months	11 (84.6%)
Svensson et al. [13]	2014	Sweden	89	1.3v16.3	7	—	82	2 (2.4%)	5.1 years	122.5 days	2 (100%)
Puapong et al. [15]	2007	US	72	0–18	11	—	61	5 (8%)	7.5 years (2 months-12years)	80% occur within 6 months	5 (100%)
Fawkner-Corbett et al. [16]	2014	UK	69	2.1–16	61	76	—	8 (12%)	—	21 days (6–51 days)	8 (100%)
Ein et al. [14]	1996	Canada	10	2–15	0	—	10	1 (10%)	6 months-13 years	2 months	1 (100%)
Pooled results	—	—	—	—	—	—	—	9.9% (4.8–20.6)	—	—	—

RCT: Randomized Control Trial.

## Length of hospital stay

4 studies reported length of hospital stay (LOS), including 1 RCT [10], 1 prospective cohort study [12], 1 retrospective [15], and 1 case control study [16]. Reports on the length of hospital stay (LOS) vary significantly across different studies (Table 2).

**Table 2.** LOS of IA and AO.

Study	Initial LOS	LOS for IA	LOS for recurrence in AO	Total LOS for IA	Total LOS for recurrence in AO	*p*
Hall et al. [10]	–	32 (26–49) hours	0 (0–23)* hours	–	–	<0.0001
Tanaka et al. [12]	13.0 ± 3.9 days	5.9 ± 2.0 days	8.7 ± 3.4 days	–	–	0.034
Fawkner–Corbett et al. [16]	8 (3–14) days	3 (1–5) days	6 (3–15) days	–	–	<0.01
Puapong et al. [15]	6.6 days	–	–	8.5 days	9.6 days	–

LOS: length of hospital stay.

* LOS for AO included all patients not only recurrence.

Among the mentioned above studies, three of them showed that LOS for IA was shorter than that for recurrent appendicitis in AO group. Both Tanaka et al. and Fawkner-Corbett et al. reported the initial LOS (13.0 ± 3.9 days; mean of 8 days), while also documenting the LOS for IA and recurrent appendicitis in AO. They found it was statistically significantly shorter in LOS for IA compared with recurrent appendicitis in AO (5.9 ± 2.0 versus 8.7 ± 3.4 days, *p* = 0.034; mean of 3 versus 6 days, *p* = <0.01) [12,16]. A retrospective cohort reported a mean total LOS of 8.5 days in IA, which was shorter than a mean total LOS of 9.6 days for recurrent appendicitis in AO [15].

In contrast, the other one had a different view that LOS for IA was longer than that for recurrence in AO [10]. Hall et al. ‘s RCT revealed there was significant difference in LOS between IA and AO (mean of 1.3 days versus 0 days, *p* = <0.001) [10]. But, in Hall et al. ‘s research, LOS for AO included all patients not only recurrence.

## Recurrence rate

Overall studies noted the recurrent appendicitis in AO group during the follow-up period (Table 1). Heterogeneity in reported recurrence rates reflects variations in the definition of recurrence, follow-up periods, and study designs among the included investigations. For patients in AO group, the recurrence rate was reported as 2.4%–34.2%. The pooled estimate of the risk of recurrence was 9.9% (CI:4.8–20.6). In most cases, the recurrent patients were treated with appendectomy.

In the RCT, 12% (6 out of 52 cases) developed histologically proven recurrent appendicitis within 1 year of follow-up. In the study by Tanaka et al. among the patients who successfully received NOT and did not undergo IA, 34.2% (13 out of 38 cases)children experienced recurrence during an average of 3.4 years of follow-up with an average recurrence time of 12.6 ± 22.3 months. In this study, the higher recurrence rate may be attributed to the inclusion of both short-term and long-term recurrent cases, with a median recurrence time of 3 months (range: 0–76 months). Moreover, the study revealed that children with recurrent appendicitis sought medical attention sooner than during their initial episode, presented with lower body temperatures, and exhibited a downward trend in white blood cell counts compared to the first occurrence.

Svensson et al. found that only 2.4% (2 out of 82 cases) patients presented with a recurrence of acute appendicitis more than 1 month after the primary event during a median follow-up of 5.1 years. Additionally, in the retrospective cohort study’s observation group, 8% (5 out of 61 cases) of patients experienced recurrence during the 7.5-year follow-up period, with 80% of these recurrences occurring within the first 6 months. Fawkner–Corbett et al’.s study identified early recurrence in 12% of cases with a mean recurrence interval of 21 days (range: 6–51 days) while patients awaited IA, thereby limiting the evaluation of long-term recurrence outcomes. Finally, a Canadian case control study, with follow-up duration extending from 6 months to 13 years, documented a recurrence rate of 10% (1 out of 10 cases) with a median recurrence time of 2 months.

## Complication rate

2 studies analyzed the complication rate in the IA and AO groups, which defined complications differently. In the RCT, a severe complication was defined as any complication requiring additional or unanticipated treatment, including, but not limited to intestinal perforation, haemorrhage requiring transfusion, wound infection requiring antibiotics, abscess formation, postoperative small bowel obstruction, and prolonged ileus (>72 h postoperatively) [10]. In the study, the percentage of complications after IA was 6% (3 out of 50 cases) while the percentage of complications after AO was 15% (2 out of 12 cases). However, one patient in IA needed further surgery due to the complication in this trial. Otherwise, another prospective series did not provide standardized criteria for complications, which included postoperative abscess, ileus, and the complications requiring readmission [12]. They showed there was no statistically significant difference in the complication rates between IA and recurrence in AO (13% versus 9%).

## Cost

Among the reviewed literature, only one study from UK surveyed cost which referred to later expenditure in IA and AO with recurrence group after initial NOT. The case-controlled study showed there was no statistically significant difference in cost between IA and recurrence in AO group (mean of £1936 versus mean of £2171, *p* = 0.09) [16].

## Operating time

Unfortunately, only one prospective cohort research evaluated operating time between IA and recurrent appendicitis required appendectomy in AO group [12]. IA demonstrated a significantly reduced operative time compared to recurrence in AO group (74.3 ± 31.2 versus 82.3 ± 21.1 mins, *p* = 0.045).

## Discussion

To date, the application of NOT, which includes the administration of antibiotics and abscess drainage, has been proved to be efficacious and stable in appendiceal abscess [9,17]. Regrettably, the researches on subsequent management following NOT remain limited and of low quality. There is still no consensus on the optimal management strategy after NOT for children presenting with appendiceal abscess. Some writers advocated IA should be advised to the patient after successful NOT, with the aim of solving the fundamental problems, avoiding recurrence and other unknown diseases [18]. Notwithstanding IA has been established as a feasible method, its efficacy is increasingly being challenged by evidence such as the low recurrence rate, postoperative complications, and potential surgical risks [19]. Therefore, a timely and thorough review of the management after NOT is essential and appropriate.

In the present study, 36.6% (145 out of 396 cases) underwent IA after 2–3months from first discharge, while 63.4% (251 out of 396 cases) accepted AO during a 0–13years follow-up period. All patients in the IA group had scheduled readmissions for surgical treatment, rendering readmission unavoidable. Unlike in the IA group, not all patients in the AO group required readmission and additional costs, despite timely follow-up visits. According to the analyzed results, the majority of reasons for readmission is recurrent appendicitis. With respect to the readmission rate, AO group is obviously lower. Furthermore, AO has a lesser impact on daily life and facilitates a quicker return to school for patients.

Additionally, after the readmission, the comparison of LOS was a crucial event for the decision. In this review, three studies proposed LOS in IA is shorter than recurrence in AO group, and two of them confirmed there is statistically significance. Conversely, a RCT thought LOS of IA was significantly longer than recurrence in AO group [10]. It’s worth mentioning that the RCT took into account LOS of all patients in AO group and calculated LOS of everyone on average. While, other studies only analyzed LOS of the patients of recurrence. Due to the variability in the analyzed participant types, careful consideration is warranted. In summary, LOS for recurrence in the AO group is longer than that in the IA group. Similarly, Darwazeh et al. analyzed 21 studies and found that LOS was longer for patients with recurrent appendicitis compared to those undergoing IA (9.6 days vs 5.0 days) [20]. Whereas, the probability of recurrent appendicitis is 2.4%-34.2%, hence, readmission and LOS could be avoided and regardless in about two-thirds or more of the patients in AO group. Furthermore, a RCT about the treatment of appendiceal abscess in adults also found that NOT alone results in shorter LOS and fewer workdays lost compared to the approach of NOT followed by IA [21].

The incidence of complication is one of the most concerned factors for surgeons in deciding which treatment to utilize, as well. Initially, NOT is recommended as the preferred treatment option, owing to its significantly lower complication rate compared to immediate appendectomy. Zhang et al. announced that initial NOT with IA was feasible for most patients with appendiceal abscess and had advantages in terms of postoperative complications, especially regarding long-term obstruction events [22]. The researchers selected the approach of NOT with IA, favored due to its lower complication rate, yet IA can’t totally eliminate the incidence of complications. Based on this notion, AO should be considered, as it allows the majority of observational patients to avoid surgery, thereby preventing complications. Besides, there was no significance difference in complication rate between IA and AO group [12]. Similarly, a systematic review in 2016 found IA and recurrence in AO showed similar complication profiles after NOT in adults [20].

Refer to healthcare cost, when taking into account all patients in the AO group, the total cost is significantly less than IA group. While if analyzing the cost between IA and recurrence in AO, the cost is similar in both groups. In addition, a retrospective research reported that cost of initial NOT was significantly less expensive than operative treatment in children with acute appendicitis, but failure of NOT would increase the total cost [23]. According to the previous studies, the success rate of NOT in the treatment of appendicitis with appendiceal abscess was higher, ranging 80% to 96.7% [23–25]. Therefore, the higher success rate of NOT and the lower recurrence rate in AO can result in the less healthcare cost in cases of appendiceal abscess.

Conventionally, the patients with recurrent appendicitis readmitted, and the majority accepted appendectomy whereas the minority insisted on NOT. In current review, 94.3% (33 out of 35 cases) underwent appendectomy after recurrence. Only one study reported that the operating time was significantly shorter in IA group than recurrence in AO group [12]. Recurrence is a new episode of the inflammation of appendix, which is characterized by acute onset and recognizable clinical signs, which is similar to the first attack. The treatment of appendectomy, in the patients of recurrent appendicitis during readmission, could be generally regarded as emergency appendectomy for the acute recurrent appendicitis. According to the previous literature reported, operating time in emergency appendectomy was longer than IA due to the inflammatory status of the appendix and adjacent tissue [26–28]. Thus, it is understandable that operative time is longer in in cases of recurrent appendicitis requiring emergency appendectomy than IA group.

This study is an infrequent review about the management after successful NOT in pediatric population. So far, in regard to comparison between IA and AO after successful NOT, there are no reviews that can be found. Little attention is paid to the management after NOT in appendiceal abscess, especially in the follow-up of the patients without IA. In present study, there are some suggestions that AO group considered as a whole may contribute to a lower readmission rate and a lower healthcare cost through reviewing. When comparing IA with recurrent appendicitis in the AO group, recurrence in the latter may result in an extended LOS and a prolonged operative time. Furthermore, patients without recurrence can avoid readmission, additional hospital stays, secondary treatments, postoperative complications, and related costs associated with recurrent appendicitis. Moreover, no significant differences in complication rates were reported between the two management strategies.

Satisfyingly, as far as we knew, there was a low incidence of recurrence in the AO group. A mixed-age meta-analysis reported that the risk of recurrence following NOT in patients with appendiceal abscess was 8.9% [29]. Besides, a prospective cohort study analyzed the risk factors might trigger recurrence and found no significant difference in gender, presence of appendicolith or abscess aspiration between patients with and without recurrence [12]. In addition, Kaminski found that the proportion of women in the recurrence group was higher [30]. A Canadian study found that the presence of appendicolith was significant for the recurrence of appendicitis [31]. Research about risk factors associated with recurrence can inform the subsequent selection of management after NOT. Further in-depth and large-sample studies are required to explore and validate relevant risk factors.

To sum up, both IA and AO represent viable treatment options following successful NOT of appendiceal abscess. IA represents an admission for appendectomy after 8 to 12 weeks from initial hospitalization and leads to shorter LOS and operating time. While complication rates and costs are similar between recurrent appendicitis in AO and IA. AO carries a quantifiable recurrence risk (2.4%–34.2%); however, patients without recurrence avoid readmission and surgery, and eliminate surgical risks. Families may make an informed choice after comprehensive counseling regarding the outcomes of IA and AO.

Notably, the paper faced several limitations, such as a limited number of studies reviewed and concerns regarding the quality of these studies. The lack of standardized outcome measures prevented meaningful quantitative comparison between studies. Furthermore, our review only included one RCT. Further randomized studies of superior quality are required to rigorously compare the diverse parameters of two distinct management methodologies. Additionally, while most studies reported the outcomes of IA, the specific consequences of AO without IA should be adequately addressed and monitored. In the future, more well-designed prospective randomized studies are required to further evaluate which management method is of greatest benefit to the children.

## Conclusion

The current study indicates that the best management strategies following a successful non-operative treatment (NOT) of appendiceal abscess remain unclear. Active observation (AO) carries a low risk of recurrence; however, non-recurrent patients can avoid readmission and reduce medical expenditures. Interval appendectomy (IA) can eliminate the risk of recurrence but necessitates a second hospitalization for surgery. Compared to recurrent surgical patients in AO, IA may show a slight trend towards a shorter LOS and operative time. Patients and families should be presented with the benefits and risks to make decisions. But these results must be interpreted with caution, as they are derived from limited evidence and a small number of supporting studies.

## Supplementary Material

Checklist.docx

## Data Availability

The data will be shared upon a reasonable request made to the corresponding author of the manuscript.
